# Identification and validation of a seven m6A-related lncRNAs signature predicting prognosis of ovarian cancer

**DOI:** 10.1186/s12885-022-09591-4

**Published:** 2022-06-08

**Authors:** Yang Song, Hui Qu

**Affiliations:** Department of Obstetrics and Gynecology, Shengjing Hospital of China Medical University, No. 36 Sanhao Street, Heping District, Shenyang, Liaoning Province 110004 P.R. China

**Keywords:** Ovarian cancer, m6A-related lncRNAs, Prognosis, Nomogram, ceRNA network

## Abstract

**Background:**

Long non-coding RNAs (lncRNAs) play an important role in angiogenesis, immune response, inflammatory response and tumor development and metastasis. m6 A (N6—methyladenosine) is one of the most common RNA modifications in eukaryotes. The aim of our research was to investigate the potential prognostic value of m6A-related lncRNAs in ovarian cancer (OC).

**Methods:**

The data we need for our research was downloaded from the Cancer Genome Atlas (TCGA) and the Gene Expression Omnibus (GEO) database. Pearson correlation analysis between 21 m6A regulators and lncRNAs was performed to identify m6A-related lncRNAs. Univariate Cox regression analysis was implemented to screen for lncRNAs with prognostic value. A least absolute shrinkage and selection operator (LASSO) Cox regression and multivariate Cox regression analyses was used to further reduct the lncRNAs with prognostic value and construct a m6A-related lncRNAs signature for predicting the prognosis of OC patients.

**Results:**

Two hundred seventy-five m6A-related lncRNAs were obtained using pearson correlation analysis. 29 m6A-related lncRNAs with prognostic value was selected through univariate Cox regression analysis. Then, a seven m6A-related lncRNAs signature was identified by LASSO Cox regression. Each patient obtained a riskscore through multivariate Cox regression analyses and the patients were classified into high-and low-risk group using the median riskscore as a cutoff. Kaplan–Meier curve revealed that the patients in high-risk group have poor outcome. The receiver operating characteristic curve revealed that the predictive potential of the m6A-related lncRNAs signature for OC was powerful. The predictive potential of the m6A-related lncRNAs signature was successfully validated in the GSE9891, GSE26193 datasets and our clinical specimens. Multivariate analyses suggested that the m6A-related lncRNAs signature was an independent prognostic factor for OC patients. Moreover, a nomogram based on the expression level of the seven m6A-related lncRNAs was established to predict survival rate of patients with OC. Finally, a competing endogenous RNA (ceRNA) network associated with the seven m6A-related lncRNAs was constructed to understand the possible mechanisms of the m6A-related lncRNAs involed in the progression of OC.

**Conclusions:**

In conclusion, our research revealed that the m6A-related lncRNAs may affect the prognosis of OC patients and identified a seven m6A-related lncRNAs signature to predict the prognosis of OC patients.

**Supplementary Information:**

The online version contains supplementary material available at 10.1186/s12885-022-09591-4.

## Background

Ovarian cancer (OC) is a common malignant tumor in gynecology. More than 70% of OC patients are diagnosed in advanced stages due to the lack of symptoms and effective screening methods in early stages [1]. Tumor cell reduction plus platinum-based chemotherapy has become the standard model for initial treatment of OC [2]. However, nearly 80% of OC patients still relapse in about three years [3]. Therefore, it is is urgent to identify new prognostic markers and therapeutic targets to promote the prognosis of OC.

At present, more than 100 RNA modification methods have been confirmed, among which m6A methylation is the most widely studied one [4]. The m6A methylation is mainly accomplished by three enzymes, including methyltransferase, demethylase and methylation recognition enzyme [5]. Recent research have shown that m6A methylation is closely related to the occurrence and development of a variety of tumors including OC. Lili Hao et al. reported that the up-regulated ALKBH5 promote the epithelial-to-mesenchymal transition (EMT) and worse outcome of uveal melanoma by inducing m6A methylation of FOXM1 [6]. Haocheng Wang et al. revealed that overexpression level of YTHDF1 was associated with the poor outcome of cervical cancer. The knockdown of YTHDF1 will inhibit the proliferation, invasion and metastasis of cervical cancer cells [7]. Le Tao et al. indicated that FTO is involved in tumorigenesis and prognosis of bladder cancer by regulating the MALAT/miR-384/MAL2 axis [8]. FTO inhibits the self-renewing of OC stem cells and the occurrence of OC by enhancing the second messenger 3', 5'-cyclic adenosine monophosphate (cAMP) signaling [9].

Long non-coding RNAs (lncRNAs) are a class of nucleotide transcripts over 200 nt in length. Studies have shown that lncRNA can regulate physiological processes such as cell differentiation, immune response and apoptosis. LncRNAs plays an important role in the occurrence and development of a variety of metabolic diseases and cancers [10]. Yanrong Lv et al. found that LncRNA TDRG1 promotes the proliferation, invasion and metastasis of breast cancer cells through miR-214-5p/CLIC4 axis [11]. The up-regulated expression level of TOPORS-AS1 inhibited the proliferation and aggressive behaviors of OC cells by disrupting the Wnt/β-catenin signaling and associated with the favorable outcome of OC patients [12]. In addition, it has been reported that several lncrnas regulate the occurrence and development of cancer through m6A modification. For example, overexpressed LINC00857 regulates the expression of E2F3 by binding to Mir-150-5p, ultimately promoting the tumogenesis and poor outcome of pancreatic cancer [13]. LNC942 promotes breast cancer cell proliferation and progression by modulating METTL14-mediated M 6A methylation [14]. LncRNA LINRIS was upregulated in colorectal cancer tissues and associated with the poor overall survival and aerobic glycolysis of colorectal cancer patients by stabilizing IGF2BP2 [15]. LncRNA GAS5-AS1 has been identified as a promoter of ALKBH5-dependent m6A demethylation in cervical cancer, thereby inhibiting the proliferation, migration and invasion of cervical cancer cells [16]. However, the potential mechanisms of m6A modifications involved in the lncRNA-dependent OC occurrence and development remains unclear. Thus, it is significant to investigate the potential mechanisms of m6A modifications of lncRNAs participated in OC.

In the current research, we identified and verified a seven m6A-related prognostic lncRNAs signature for predicting the prognosis of OC based on the data obtained from the Cancer Genome Atlas (TCGA) and the Gene Expression Omnibus (GEO) database. Each patient obtained a riskscore and the patients were classified into high-and low-risk group using the median value. The patients in low-risk group were associated with favorable prognosis. The receiver operating characteristic (ROC) curve revealed that the predictive potential of the m6A-related lncRNAs signature for OC was powerful. The predictive potential of the m6A-related lncRNAs signature was successfully validated in the GSE9891, GSE26193 datasets and 60 clinical specimens. A nomogram was constructed based on the expression level of the seven m6A-related lncRNAs to predict the survival rate of OC patients. Finally, a ceRNA network related to the seven m6A-related lncRNAs was established.

## Materials and methods

### Data acquisition

The training cohort, TCGA-OV dataset containing 379 patients were downloaded from the Genomic Data Commons Data Portal (https://portal.gdc.cancer.gov/). The validation cohort, GSE9891 [20] and GSE26193 [21] dataset including 285 and 107 patients respectively were acquired from Gene Expression Omnibus (GEO) database (https://www.ncbi.nlm.nih.gov/). Perl software was used for data integration and extraction of lncRNAs expression data and corresponding clinical data.

### Specimen collection

A total of 60 OC samples were collected at ShengJing Hospital of China Medical University (Shenyang, China) from January to December 2015. Clinical information of the OC samples is presented in Supplementary Table 1. The inclusion criterion of the samples was as follows: (1) High-grade serous OC diagnosed by postoperative pathology; (2) All patients underwent surgical treatment, and the lesion tissue was retained during the operation; (3) Complete prognostic information was available (4) Informed and consented participants in this study. The exclusion criteria of the samples was as follows: (1) Patients with cognitive dysfunction and autoimmune system diseases; (2) The patient had received hormonal or chemoradiotherapy prior to tissue collection; (3) Complicated with mental abnormalities, tumors in other parts or severe liver and kidney function abnormalities. This study was approved by the ethics committee of the ShengJing Hospital of China Medical University, and informed consent was obtained from all patients. In addition, all methods were executed in accordance with relevant guidelines and regulations.

### Identification of the m6A-related lncRNAs

The 21 m6A regulators including 8 writers (METTL3, ZC3H13, METTL14, RBM15B, CBLL1, WTAP, RBM15, and KIAA1429), 2 erasers (FTO and ALKBH5), and 11 readers (YTHDC1, YTHDC2, ELAVL1, YTHDF1, LRPPRC, YTHDF2, FMR1, YTHDF3, HNRNPC, HNRNPA2B1, and IGF2BP1) were extracted from the TCGA-OV dataset [22]. Pearson correlation analysis was performed to calculate the correlation coefficient between 21 m6A regulators and lncRNAs. LncRNAs meet the screening criteria *p* < 0.001 and |R|> 0.4 were considered as m6A-related lncRNAs [23]. A lncRNAs- m6A regulators network was constructed and visualized by cytoscope software. Finally, function enrichment analysis was used to explore the functions of the m6A regulators in the network involed in OC through GenCLiP310 online website (http://ci.smu.edu.cn/genclip3/analysis.php) [24].

### Construction of the m6A-related lncRNAs prognostic signature

The m6A-related lncRNAs were fitted into univariate Cox regression analysis to obtain the m6A-related lncRNAs with prognostic value according to *p* < 0.05. A least absolute shrinkage and selection operator (LASSO) Cox regression and forward stepwise method was conducted to further narrow the prognostic related lncRNAs. Multivariate Cox regression analysis was implemented to calculate the regression coefficients of the selected m6A-related lncRNAs. Each patient acquired a riskscore according to the formula:

Risk score = ∑Coef_i_ * x_i_ (Coef_i_ represents the regression coefficient, x_i_ represents the expression level of m6A-related lncRNAs).

### Real-time qPCR

Total RNA of OC samples was extracted using TriZol Reagent (Takara, Japan). cDNA synthesis was carried out using the AMV reverse transcriptase reagent box (Takara, Japan). Real-time PCR was performed using a 2 × SYBR Green PCR Master Mix. Next, the 2-ΔΔCt method was used to calculate the relative gene expression with GAPDH serving as an internal reference. The sequences of primers used for RT-qPCR are presented in Supplementary Table 2.

### Evaluation and validation of the m6A-related lncRNAs prognostic signature

Patients were divided into high- and low- risk groups based on the median riskscore. Kaplan–Meier (K-M) method was used to compare the differences in prognosis between groups. The receiver-operating characteristics (ROC) curve was performed to evaluate the effectiveness of the m6A-related lncRNAs prognostic signature and the area under the curve (AUC) was calculated. We then validated the results in GSE9891 and GSE26193 dataset. Finally, multivariate Cox regression analysis was used to investigate whether the riskscore was independent of the clinicopathological parameters as an independent prognostic factor in OC patients.

### Construction of the nomogram model

To predict the survival rate of the OC patients, a nomogram model was conducted based on the expression level of the m6A-related lncRNAs prognostic signature using the “rms”package in R software. Calibration curves at 1-,3-,5- year were drawn to assess the consistency between actual and predicted survival rates [25].

### Construction of the ceRNA network related to the m6A-related lncRNAs prognostic signature

CeRNA network plays an important role in the occurrence and progression of ovarian cancer. We constructed a ceRNA network related to the m6A-related lncRNAs prognostic signature and the corresponding m6A regulators. Firstly, we obtained the miRNAs interacted with the m6A-related lncRNAs prognostic signature from the miRDB online website (http://mirdb.org/custom.html) [26]. We then acquired the possible miRNAs interacted with the m6A regulators from miRWalk online website (http://mirwalk.umm.uni-heidelberg.de/) [27]. After intersecting the predicted miRNAs, a lncRNAs—miRNAs—mRNAs ceRNA network was conducted and visualized by cytoscope software.

## Results

### Construction of the seven m6A-related lncRNAs prognostic signature

**Fig. 1:**
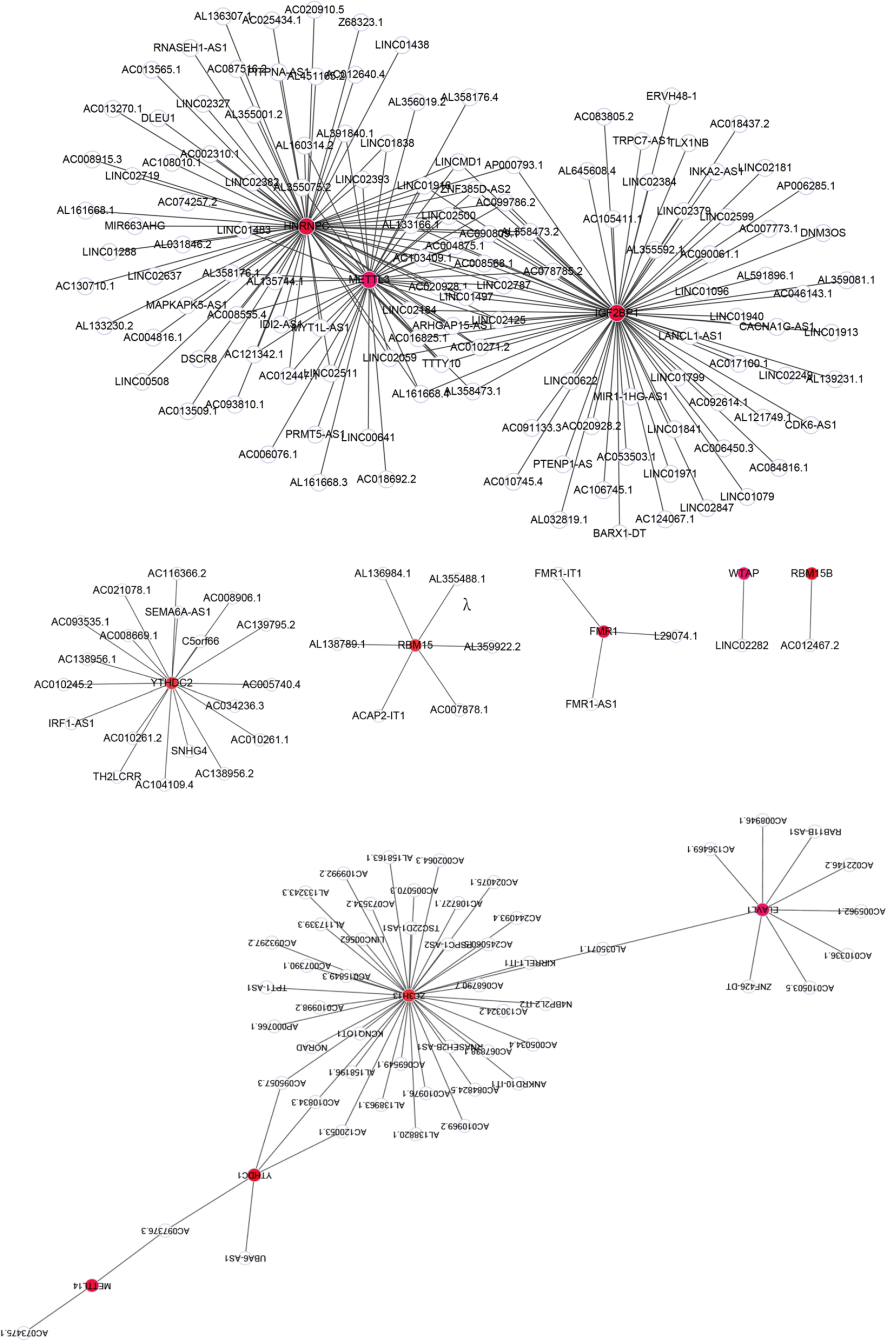
The network between 275 lncRNAs- 12 m6A regulators. Red represents m6A regulators, white represents m6A-related lncRNAs

**Fig. 2:**
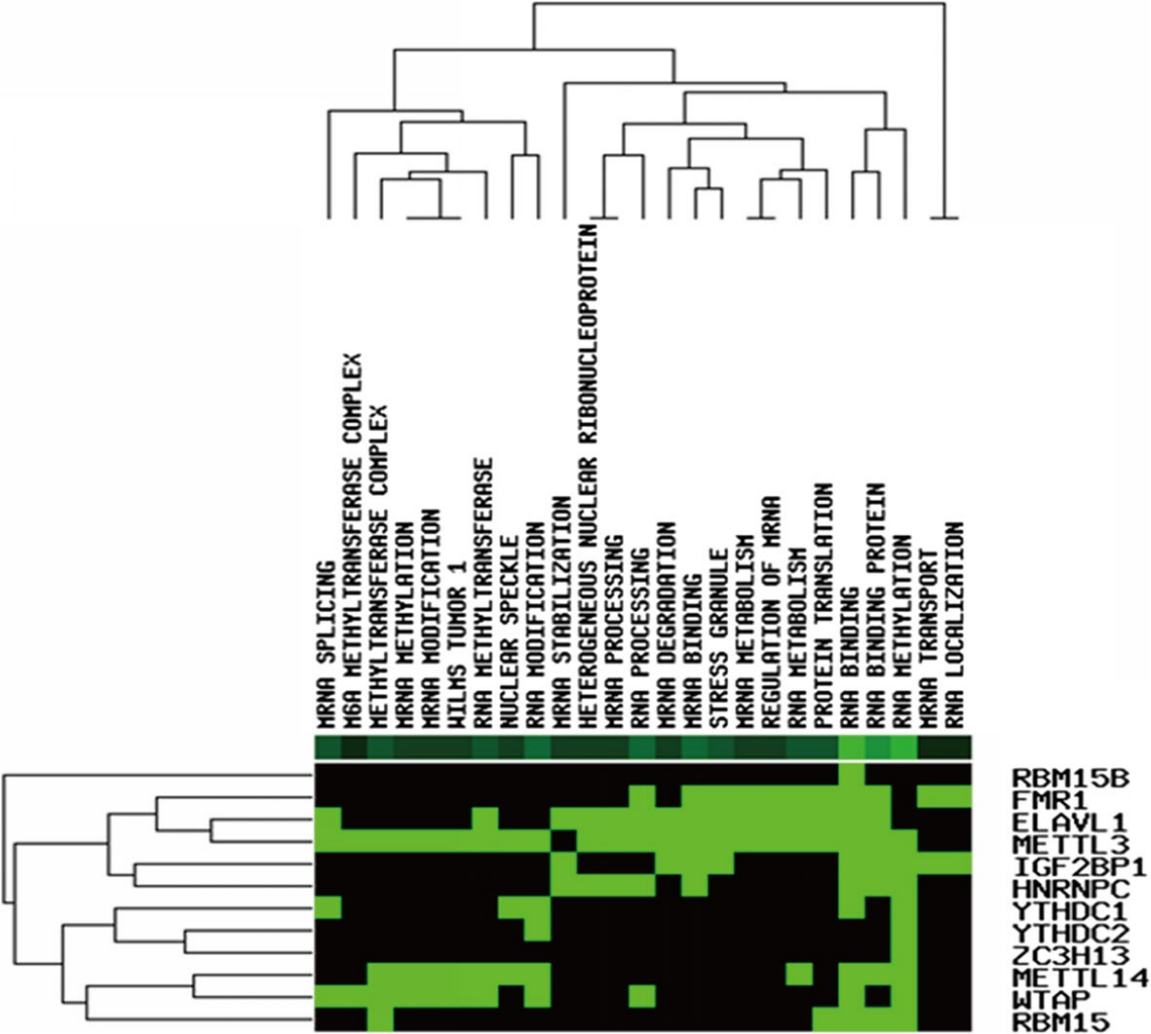
Function enrichment analysis involved in the 12 m6A regulators in the ceRNA network

**Fig. 3:**
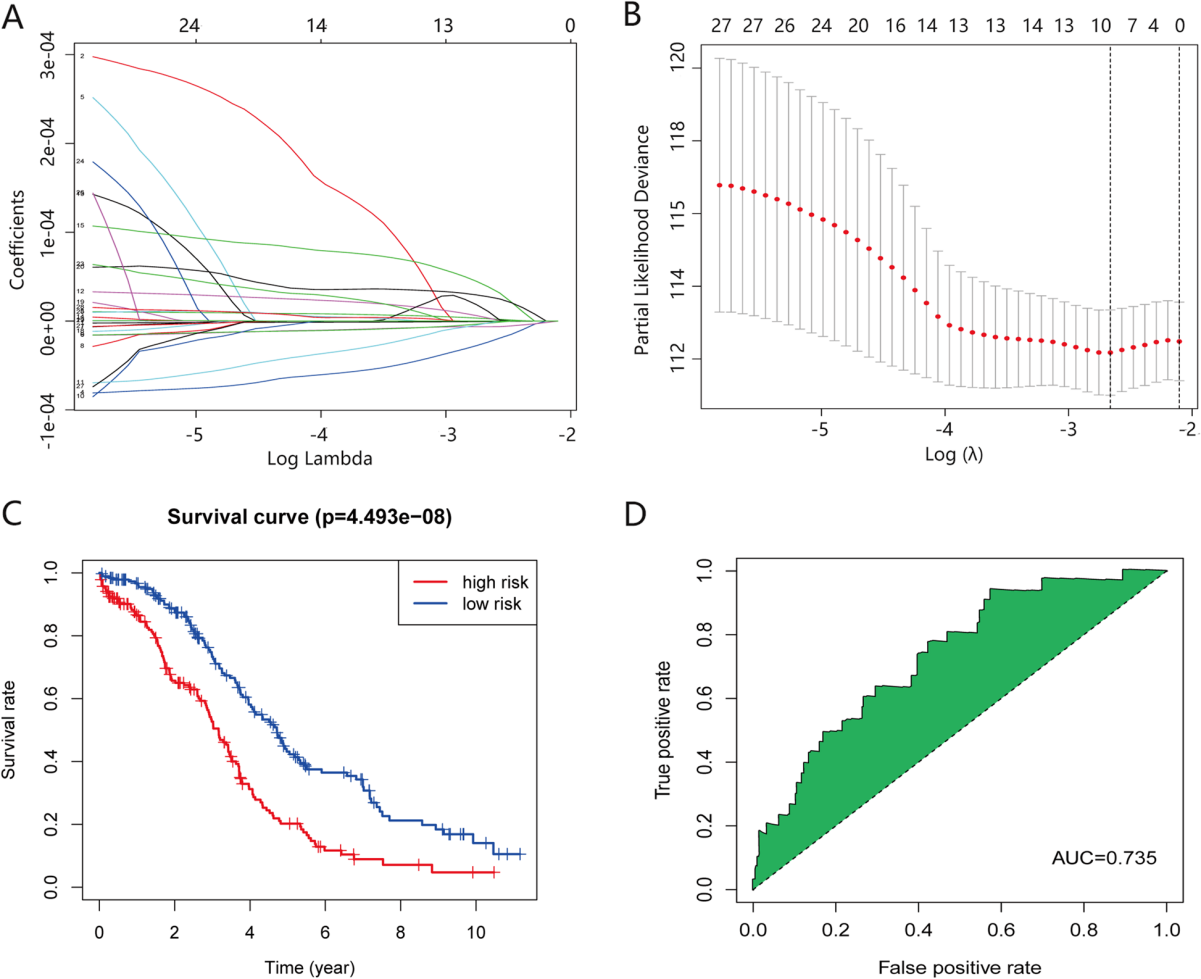
Identification of a seven m6A-related lncRNAs prognostic signature. **A** LASSO coefficient profiles of the 29 m6A-related lncRNAs. **B** The penalization coefficient λ in the LASSO model was tuned using tenfold cross-validation and the minimum criterion. AUC metrics (y-axis) were plotted against log(λ) (bottom x-axis). The top x-axis indicates the number of predictors for the given log (λ). For the optimal λ, ten m6A-related lncRNAs with non-zero coefficient were selected. **C** K-M survival curve showed survival analysis of the seven m6A-related lncRNAs prognostic signature in TCGA-OV dataset. **D** ROC curve analysis of the seven m6A-related lncRNAs prognostic signature in TCGA-OV dataset

We obtained 275 m6A-related lncRNAs based on the pearson correlation analysis between the 21 m6A regulators and lncRNAs (Table1). 275 lncRNAs- 12 m6A regulators network was constructed and visualized by cytoscope software (Fig. 1). The 12 m6A regulators in the network was fitted into the GenCLiP310 online website to explore the function involed in OC. The results indicated that the 12 m6A regulators involved in OC progression through post-transcriptional modification of RNA (Fig. 2). 29 m6A-related lncRNAs with prognostic value for OC patients selected from the 275 m6A-related lncRNAs using univariate Cox regression analysis (Table 2, *p* < 0.05). The expression level of 29 m6A-related lncRNAs and corresponding overall survival, survival status of patients were substituted into Lasso regression analysis to select and shrink the variables.Ten m6A-related lncRNAs prognostic signature (AC004816.1, AC013270.1, AL138820.1, AC008669.1, AC010336.1, AC097376.3, AC130710.1, ACAP2-IT1, AL138820.1 and CACNA1G-AS1) was selected according to the optimal λ value of tenfold cross-validation (Fig. 3A and B). Forward stepwise method was conducted to further narrow the prognostic related lncRNAs. A seven m6A-related lncRNAs prognostic signature (AC008669.1, AC010336.1, AC097376.3, AC130710.1, ACAP2-IT1, AL138820.1 and CACNA1G-AS1) was obtained. Multivariate Cox regression analysis was implemented to calculate the regression coefficients of the seven m6A-related lncRNAs prognostic signature (Table 3). Each patient acquired a riskscore according to the formula: riskscore = 9.84E-06* exp AC008669.1 -7.82E-05 * exp AC010336.1 -1.56E-05* exp AC097376.3 -4.26E-05* exp AC130710.1 + 3.19E-05* exp ACAP2-IT1 + 0.00011047* exp AL138820.1 + 5.74E-05 * exp CACNA1G-AS1.

**Table1:** Correlation between m6A regulators and lncRNAs in ovarian cancer

GENE1	GENE2	P	R
AC083805.2	IGF2BP1	7.87E-51	0.670561
AL356019.2	METTL3	4.92E-44	0.634181
AC091133.3	IGF2BP1	2.45E-43	0.630157
PRMT5-AS1	METTL3	2.95E-43	0.629683
LINC01096	IGF2BP1	3.19E-42	0.623587
AL591896.1	IGF2BP1	1.09E-39	0.608062
AL355075.2	METTL3	7.12E-34	0.568839
LINC01838	METTL3	9.45E-34	0.567947
LINC01971	IGF2BP1	2.66E-32	0.557248
AL138963.1	ZC3H13	9.31E-32	0.553138
AC092614.1	IGF2BP1	9.32E-32	0.553134
AC005070.3	ZC3H13	1.03E-31	0.552793
AC010271.2	HNRNPC	1.53E-31	0.551494
AL160314.2	METTL3	3.70E-31	0.548539
LINC01841	IGF2BP1	2.57E-30	0.541948
TPT1-AS1	ZC3H13	3.75E-30	0.540651
AL135744.1	HNRNPC	1.13E-29	0.536832
AC020928.1	IGF2BP1	1.99E-29	0.534844
AC008946.1	ELAVL1	2.87E-29	0.533544
AC090809.1	IGF2BP1	3.81E-29	0.532544
AC008915.3	HNRNPC	9.09E-29	0.529446
AC116366.2	YTHDC2	1.26E-28	0.528284
RAB11B-AS1	ELAVL1	1.46E-28	0.52774
AL121749.1	IGF2BP1	1.53E-28	0.527572
RNASEH1-AS1	HNRNPC	6.72E-28	0.522191
LINC02500	HNRNPC	5.00E-27	0.514738
AC021078.1	YTHDC2	1.41E-26	0.510825
AC022146.2	ELAVL1	1.44E-26	0.510739
AC005034.4	ZC3H13	1.50E-26	0.510571
AL161668.4	HNRNPC	1.76E-26	0.50996
AL133166.1	HNRNPC	2.23E-26	0.50907
AL358473.1	IGF2BP1	2.41E-26	0.508762
LINC01799	IGF2BP1	4.13E-26	0.506687
AC010834.3	ZC3H13	2.25E-25	0.500076
ZNF385D-AS2	HNRNPC	2.33E-25	0.49994
AC004875.1	HNRNPC	3.40E-25	0.498435
AL391840.1	HNRNPC	3.55E-25	0.498267
LINC02184	HNRNPC	4.37E-25	0.49744
LINC01497	HNRNPC	5.60E-25	0.496455
LINC02393	HNRNPC	7.85E-25	0.495101
LINCMD1	HNRNPC	8.27E-25	0.49489
AC099786.2	HNRNPC	9.32E-25	0.494409
AL161668.3	METTL3	1.01E-24	0.494076
LINC01916	HNRNPC	1.05E-24	0.493918
AC016825.1	IGF2BP1	1.25E-24	0.493237
AL032819.1	IGF2BP1	1.26E-24	0.493193
AC008906.1	YTHDC2	1.31E-24	0.493032
AP000766.1	ZC3H13	1.39E-24	0.492789
AC008555.4	HNRNPC	1.42E-24	0.492716
AC020928.2	IGF2BP1	2.02E-24	0.491281
AC020928.1	HNRNPC	2.14E-24	0.491041
LINC02181	IGF2BP1	2.67E-24	0.490155
LINC01497	IGF2BP1	2.84E-24	0.489899
AC010271.2	METTL3	3.12E-24	0.489514
AL359081.1	IGF2BP1	3.48E-24	0.489061
MYT1L-AS1	HNRNPC	5.55E-24	0.487147
AL355592.1	IGF2BP1	5.86E-24	0.486926
TTTY10	HNRNPC	7.48E-24	0.485916
AL161668.4	METTL3	7.55E-24	0.485879
LINC00562	ZC3H13	8.01E-24	0.485633
AC106745.1	IGF2BP1	8.86E-24	0.485215
AC121342.1	HNRNPC	1.19E-23	0.48401
AL133166.1	METTL3	1.44E-23	0.483187
LINC02059	HNRNPC	1.78E-23	0.482318
MIR1-1HG-AS1	IGF2BP1	1.84E-23	0.482176
C5orf66	YTHDC2	1.84E-23	0.482167
MIR663AHG	HNRNPC	1.90E-23	0.482037
IDI2-AS1	METTL3	2.00E-23	0.481815
AC010503.5	ELAVL1	2.06E-23	0.481695
AC108727.1	ZC3H13	3.00E-23	0.480126
AP000793.1	IGF2BP1	3.19E-23	0.479858
LINC02500	METTL3	3.20E-23	0.479848
LINC02599	IGF2BP1	7.36E-23	0.476316
AL135744.1	METTL3	7.98E-23	0.475966
LINCMD1	IGF2BP1	1.13E-22	0.474486
ZNF385D-AS2	METTL3	1.16E-22	0.474355
LINCMD1	METTL3	1.35E-22	0.473699
AL139231.1	IGF2BP1	2.52E-22	0.471022
LINC01916	METTL3	2.95E-22	0.470334
AC105411.1	IGF2BP1	3.34E-22	0.469788
AC087516.2	HNRNPC	3.64E-22	0.469412
LINC02847	IGF2BP1	4.60E-22	0.468394
ERVH48-1	IGF2BP1	5.75E-22	0.467409
LINC02379	IGF2BP1	8.48E-22	0.465697
AC099786.2	IGF2BP1	9.18E-22	0.465349
FMR1-AS1	FMR1	9.31E-22	0.465285
UBA6-AS1	YTHDC1	1.11E-21	0.464513
AC103409.1	HNRNPC	1.24E-21	0.464024
AC046143.1	IGF2BP1	1.38E-21	0.463532
SEMA6A-AS1	YTHDC2	1.39E-21	0.463524
AC130710.1	HNRNPC	1.59E-21	0.462904
AC010745.4	IGF2BP1	1.63E-21	0.462792
LINC02184	METTL3	2.77E-21	0.460417
KCNQ1OT1	ZC3H13	2.81E-21	0.460351
AC010261.2	YTHDC2	3.42E-21	0.45947
AC097376.3	YTHDC1	3.58E-21	0.459273
TTTY10	METTL3	4.14E-21	0.458614
LINC01940	IGF2BP1	4.26E-21	0.45848
AC084824.5	ZC3H13	4.78E-21	0.457957
LINC01079	IGF2BP1	5.82E-21	0.457066
AC012447.1	HNRNPC	6.12E-21	0.45684
LINC01438	HNRNPC	6.34E-21	0.456674
AC084816.1	IGF2BP1	6.72E-21	0.456411
AL117339.3	ZC3H13	7.15E-21	0.456131
AC104109.4	YTHDC2	7.46E-21	0.455936
LINC00508	HNRNPC	8.08E-21	0.455568
MAPKAPK5-AS1	HNRNPC	8.12E-21	0.455547
AC007773.1	IGF2BP1	8.21E-21	0.455499
ARHGAP15-AS1	HNRNPC	8.50E-21	0.45534
LINC02511	HNRNPC	9.54E-21	0.45481
FMR1-IT1	FMR1	9.60E-21	0.454784
AC008568.1	HNRNPC	1.11E-20	0.454128
TSC22D1-AS1	ZC3H13	1.74E-20	0.452037
LINC01483	HNRNPC	1.83E-20	0.451804
AC053503.1	IGF2BP1	1.95E-20	0.451515
LINC02249	IGF2BP1	1.99E-20	0.451425
AC121342.1	METTL3	2.28E-20	0.450798
AC004875.1	METTL3	2.98E-20	0.449547
ARHGAP15-AS1	IGF2BP1	3.17E-20	0.449267
AC120053.1	ZC3H13	3.64E-20	0.448619
ZNF385D-AS2	IGF2BP1	3.91E-20	0.448277
AC136469.1	ELAVL1	4.93E-20	0.447196
AL136984.1	RBM15	5.45E-20	0.446725
DNM3OS	IGF2BP1	5.69E-20	0.446515
LINC02125	HNRNPC	5.97E-20	0.446295
SNHG4	YTHDC2	6.17E-20	0.446134
AL358176.1	METTL3	6.84E-20	0.445653
AL355001.2	HNRNPC	7.45E-20	0.445247
LINC02184	IGF2BP1	8.21E-20	0.444787
AL138820.1	ZC3H13	8.69E-20	0.444521
CACNA1G-AS1	IGF2BP1	9.04E-20	0.444332
AL133166.1	IGF2BP1	1.17E-19	0.443097
AC010336.1	ELAVL1	1.25E-19	0.44279
AL161668.4	IGF2BP1	1.26E-19	0.442738
AC090809.1	HNRNPC	1.37E-19	0.442343
LINC01288	HNRNPC	1.44E-19	0.442121
AC120053.1	YTHDC1	1.48E-19	0.441979
LINC01916	IGF2BP1	1.58E-19	0.441666
LINC02059	IGF2BP1	1.80E-19	0.441046
AC007390.1	ZC3H13	1.97E-19	0.440626
ANKRD10-IT1	ZC3H13	1.98E-19	0.440582
CDK6-AS1	IGF2BP1	1.99E-19	0.440562
AC095057.3	ZC3H13	2.02E-19	0.440503
AC006076.1	METTL3	2.35E-19	0.439768
AC069549.1	ZC3H13	2.77E-19	0.438982
LINC02125	METTL3	3.10E-19	0.438438
LINC00622	IGF2BP1	3.57E-19	0.437746
AC093810.1	HNRNPC	3.69E-19	0.437586
LINC02382	HNRNPC	3.73E-19	0.437539
LINC02059	METTL3	3.96E-19	0.437241
ARHGAP15-AS1	METTL3	4.44E-19	0.436689
AL451165.2	HNRNPC	4.83E-19	0.436276
AC074257.2	HNRNPC	6.13E-19	0.435117
AL358473.2	IGF2BP1	6.79E-19	0.434618
AL391840.1	METTL3	6.95E-19	0.434501
AC004875.1	IGF2BP1	7.05E-19	0.434434
AL158163.1	ZC3H13	7.20E-19	0.434329
TTTY10	IGF2BP1	7.67E-19	0.43402
LINC01497	METTL3	8.89E-19	0.433294
AC093297.2	ZC3H13	9.03E-19	0.43322
LINC02787	HNRNPC	1.12E-18	0.432163
AC012640.4	HNRNPC	1.16E-18	0.431985
LINC02500	IGF2BP1	1.16E-18	0.431964
AC139795.2	YTHDC2	1.28E-18	0.431484
AL358473.1	HNRNPC	1.31E-18	0.431377
BARX1-DT	IGF2BP1	1.43E-18	0.430957
AC090061.1	IGF2BP1	1.49E-18	0.430735
AC008568.1	METTL3	1.50E-18	0.430723
AL035071.1	ZC3H13	1.68E-18	0.430135
MYT1L-AS1	METTL3	1.86E-18	0.429646
L29074.1	FMR1	1.86E-18	0.429637
AC020910.5	HNRNPC	1.87E-18	0.429611
LINC01913	IGF2BP1	1.92E-18	0.429483
LINC01838	HNRNPC	1.95E-18	0.429417
ZNF426-DT	ELAVL1	2.09E-18	0.429055
AC109992.2	ZC3H13	2.48E-18	0.428215
AL358176.1	HNRNPC	2.50E-18	0.428177
AC073475.1	METTL14	2.56E-18	0.428041
KIRREL1-IT1	ZC3H13	2.74E-18	0.427716
LINC02787	IGF2BP1	2.77E-18	0.427654
AL136307.1	HNRNPC	2.91E-18	0.427399
IRF1-AS1	YTHDC2	3.06E-18	0.42715
AC005962.1	ELAVL1	3.15E-18	0.427015
AC099786.2	METTL3	3.27E-18	0.426819
AL358473.2	HNRNPC	3.43E-18	0.426585
AC016825.1	HNRNPC	3.82E-18	0.426041
AC078785.2	IGF2BP1	4.52E-18	0.425188
AC013270.1	HNRNPC	5.46E-18	0.424238
AL359922.2	RBM15	5.88E-18	0.423862
AC010271.2	IGF2BP1	5.95E-18	0.423796
IDI2-AS1	HNRNPC	6.00E-18	0.423758
AC017100.1	IGF2BP1	6.74E-18	0.423167
NORAD	ZC3H13	6.91E-18	0.423037
AP000793.1	HNRNPC	7.94E-18	0.422332
AC005740.4	YTHDC2	8.02E-18	0.422283
TH2LCRR	YTHDC2	8.37E-18	0.422059
LINC02282	WTAP	9.05E-18	0.421661
AC010834.3	YTHDC1	9.74E-18	0.421285
LINC01483	METTL3	9.97E-18	0.421168
AL160314.2	HNRNPC	1.28E-17	0.419904
AL158196.1	ZC3H13	1.33E-17	0.419678
PITPNA-AS1	HNRNPC	1.38E-17	0.419506
AC013509.1	HNRNPC	1.40E-17	0.419439
AC007878.1	RBM15	1.40E-17	0.419407
AC138956.1	YTHDC2	1.61E-17	0.418714
AC012467.2	RBM15B	1.62E-17	0.418681
AC124067.1	IGF2BP1	1.76E-17	0.418235
AC004816.1	HNRNPC	2.16E-17	0.417181
AC245060.5	ZC3H13	2.23E-17	0.417028
Z68323.1	HNRNPC	2.44E-17	0.416548
PSPC1-AS2	ZC3H13	2.62E-17	0.416173
AC010261.1	YTHDC2	2.63E-17	0.416159
AL138789.1	RBM15	2.91E-17	0.415633
AC020928.1	METTL3	3.07E-17	0.41536
AL645608.4	IGF2BP1	3.08E-17	0.415333
AC018437.2	IGF2BP1	3.13E-17	0.415248
LINC00641	METTL3	3.22E-17	0.415102
AC103409.1	METTL3	3.29E-17	0.414998
AC010976.1	ZC3H13	3.36E-17	0.414879
AL161668.1	HNRNPC	3.40E-17	0.414819
AC012447.1	METTL3	3.72E-17	0.414356
AP006285.1	IGF2BP1	3.79E-17	0.414249
TRPC7-AS1	IGF2BP1	4.26E-17	0.413638
N4BP2L2-IT2	ZC3H13	4.36E-17	0.413511
AL358176.4	METTL3	4.46E-17	0.413403
AC025434.1	HNRNPC	4.70E-17	0.413121
AC097376.3	METTL14	4.99E-17	0.412807
AC138956.2	YTHDC2	5.02E-17	0.412772
AC244093.4	ZC3H13	5.52E-17	0.412278
AC068790.7	ZC3H13	5.63E-17	0.412173
LINC02787	METTL3	5.79E-17	0.41202
AC006450.3	IGF2BP1	7.00E-17	0.411019
AL133243.3	ZC3H13	7.70E-17	0.410517
AC008555.4	METTL3	8.20E-17	0.410178
LINC02384	IGF2BP1	8.24E-17	0.410157
LINC02125	IGF2BP1	8.97E-17	0.409705
AC008568.1	IGF2BP1	9.01E-17	0.409678
TLX1NB	IGF2BP1	9.10E-17	0.40963
AC010998.2	ZC3H13	9.97E-17	0.40914
AC095057.3	YTHDC1	1.00E-16	0.409099
AC015849.3	ZC3H13	1.01E-16	0.409095
DSCR8	HNRNPC	1.01E-16	0.409085
AC008669.1	YTHDC2	1.11E-16	0.408546
AC093535.1	YTHDC2	1.16E-16	0.408333
INKA2-AS1	IGF2BP1	1.17E-16	0.408287
LINC02637	HNRNPC	1.21E-16	0.408088
AC078785.2	METTL3	1.27E-16	0.407856
ACAP2-IT1	RBM15	1.31E-16	0.407682
DLEU1	HNRNPC	1.36E-16	0.407474
AC002064.3	ZC3H13	1.37E-16	0.407456
LINC02327	HNRNPC	1.42E-16	0.407269
LINC02382	METTL3	1.50E-16	0.40694
AC013565.1	HNRNPC	1.51E-16	0.406933
LINC02719	HNRNPC	1.51E-16	0.406925
AC130324.2	ZC3H13	1.60E-16	0.406616
AC067838.1	ZC3H13	1.66E-16	0.406404
LINC02393	METTL3	1.71E-16	0.406237
AC024075.1	ZC3H13	1.81E-16	0.405933
AC002310.1	HNRNPC	2.15E-16	0.405007
AC018692.2	METTL3	2.22E-16	0.404837
AL035071.1	ELAVL1	2.36E-16	0.404518
AL355488.1	RBM15	2.46E-16	0.404286
AC010245.2	YTHDC2	2.74E-16	0.403704
AC034236.3	YTHDC2	2.95E-16	0.403307
AC103409.1	IGF2BP1	3.79E-16	0.401942
LANCL1-AS1	IGF2BP1	3.88E-16	0.401807
AC090809.1	METTL3	4.03E-16	0.401607
RNASEH2B-AS1	ZC3H13	4.11E-16	0.401492
AC010969.2	ZC3H13	4.17E-16	0.401421
PTENP1-AS	IGF2BP1	4.22E-16	0.401356
LINC02511	METTL3	4.26E-16	0.401302
AC073534.2	ZC3H13	5.16E-16	0.400244
AL031846.2	HNRNPC	3.45E-16	-0.40244
AC108010.1	HNRNPC	7.51E-17	-0.41064
AL133230.2	HNRNPC	4.70E-17	-0.41312

**Table 2:** 29 m6A-related lncRNAs with prognostic value for OC patients selected by Univariate Cox regression analysis

id	HR	HR.95L	HR.95H	*p* value
CACNA1G-AS1	1.000052	1.000028	1.000076	2.57E-05
AL121749.1	1.000133	1.000057	1.000209	0.000574
LINC02599	1.000061	1.000025	1.000097	0.000862
LINC02847	1.00006	1.000023	1.000097	0.001387
LINC01940	1.000341	1.000121	1.000562	0.002403
DNM3OS	1.000003	1.000001	1.000005	0.00381
AC008669.1	1.00001	1.000003	1.000018	0.004955
AL355592.1	1.000013	1.000004	1.000023	0.00582
AC004816.1	0.999996	0.999994	0.999999	0.01277
AL138820.1	1.000146	1.00003	1.000262	0.013608
AC106745.1	1.000024	1.000005	1.000043	0.01448
ACAP2-IT1	1.000024	1.000004	1.000043	0.016816
AC013270.1	0.999974	0.999952	0.999995	0.017511
AC084816.1	1.000134	1.000022	1.000247	0.019147
AC006450.3	1.000341	1.000056	1.000627	0.019222
AC010336.1	0.999903	0.999822	0.999985	0.01975
AL451165.2	0.999999	0.999997	1	0.021466
AC010745.4	1.000154	1.000021	1.000287	0.02312
AL591896.1	1.000021	1.000003	1.000039	0.023306
AC097376.3	0.999986	0.999974	0.999998	0.026463
AP006285.1	1.00011	1.000013	1.000207	0.026714
AC130710.1	0.999955	0.999915	0.999995	0.026845
ZNF426-DT	0.999998	0.999997	1	0.030997
AL135744.1	0.999979	0.999959	0.999998	0.033115
MIR1-1HG-AS1	1.000045	1.000003	1.000086	0.035192
LINC01971	1.000035	1.000002	1.000067	0.038928
AC083805.2	1.000023	1.000001	1.000046	0.044915
LINC01096	1.000008	1	1.000016	0.046686
LINC01497	1.000016	1	1.000031	0.048521

**Table 3:** The 7 m6A regulators -related lncRNAs significantly associated with the OS of ovarian cancer

id	coef	HR	HR.95L	HR.95H	*p* value
AC008669.1	9.84E-06	1.00001	1.000002	1.000018	0.018953
AC010336.1	-7.82E-05	0.999922	0.999838	1.000006	0.068191
AC097376.3	-1.56E-05	0.999984	0.999972	0.999997	0.015893
AC130710.1	-4.26E-05	0.999957	0.999918	0.999997	0.036352
ACAP2-IT1	3.19E-05	1.000032	1.000012	1.000052	0.001653
AL138820.1	0.00011047	1.00011	0.999978	1.000243	0.103144
CACNA1G-AS1	5.74E-05	1.000057	1.000033	1.000081	2.92E-06

### Evaluation and validation of the seven m6A-related lncRNAs prognostic signature

**Fig. 4:**
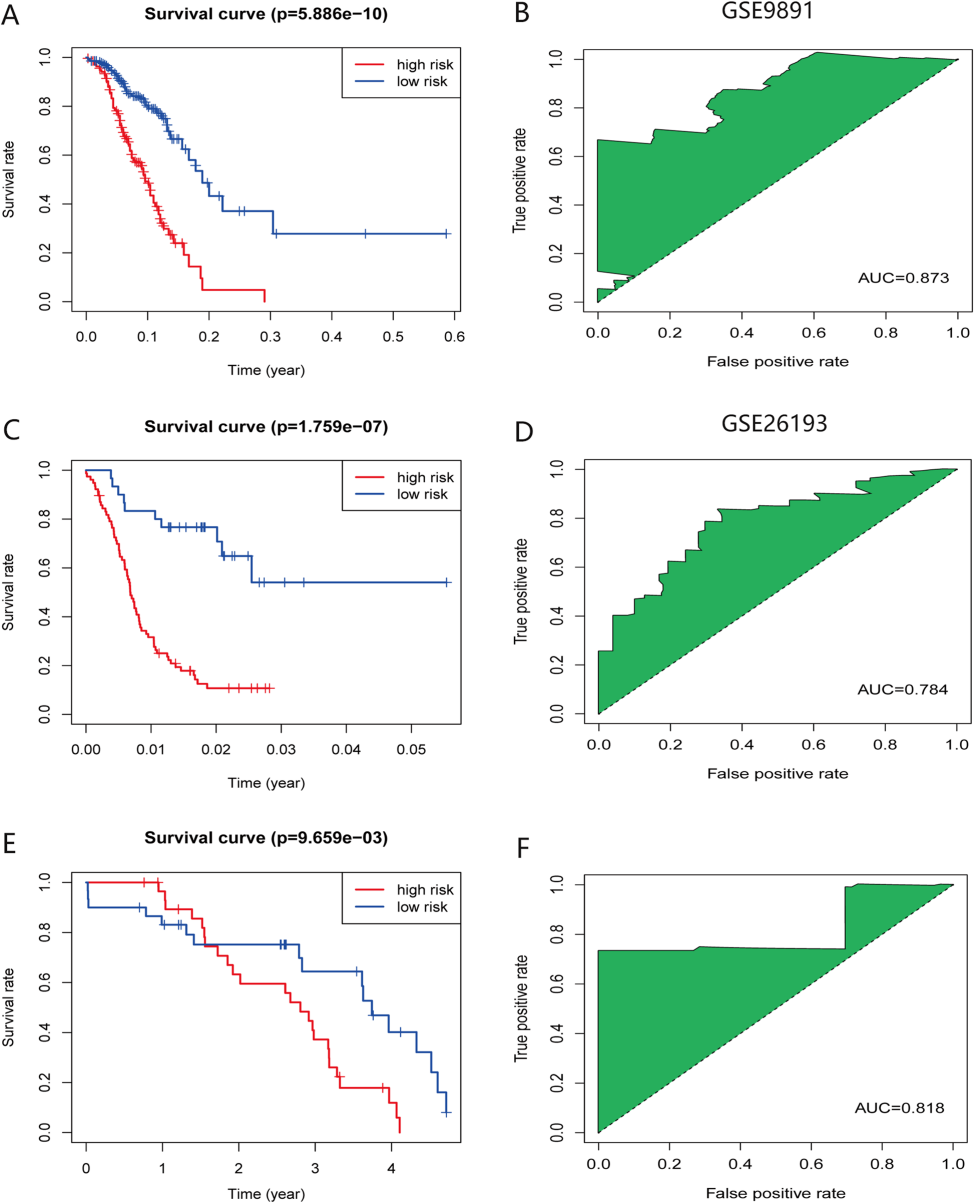
Evaluation of the seven m6A-related lncRNAs prognostic signature in GSE9891 and GSE26193 dataset. **A** K-M survival curve showed survival analysis of the m6A-related lncRNAs prognostic signature in GSE9891 dataset. **B** ROC curve analysis of the m6A-related lncRNAs prognostic signature in GSE9891 dataset. **C** K-M survival curve showed survival analysis of m6A-related lncRNAs prognostic signature in GSE26193 dataset. **D** ROC curve analysis of the m6A-related lncRNAs prognostic signature in GSE26193 dataset. **E** The K-M survival curve showed survival analysis of m6A-related lncRNAs prognostic signature in in our clinical specimens. **F** ROC curve analysis of of the m6A-related lncRNAs prognostic signature in our clinical specimens

Patients were divided into high- and low- risk groups based on the median riskscore. K-M curve indicated that the patients in high-risk group have poor prognosis cpmpared with the patients in low- risk group (Fig. 3C, *p* = 4.493e-08). The ability of the seven m6A-related lncRNAs prognostic signature to predict patient outcome was evaluated by a ROC curve, and the AUC value was 0.735 (Fig. 3D). To verify the the seven m6A-related lncRNAs prognostic signature established in TCGA database, the riskscore of patients in GSE9891, GSE26193 dataset as well as 60 clinical specimens was calculated based on the seven m6A-related lncRNAs. The patients were divided into high- and low- risk group according to the median riskscore. Similar to the results obtained in the TCGA database, patients in the high-risk group had a shorter overall survival compared to patients in the low-risk group (Fig. 4A, C and E). The ROC curve was drawn in GSE9891, GSE26193 dataset as well as 60 clinical specimens, the AUC value was 0.873, 0.784 and 0.818, respectively (Fig. 4B, D and F).

### Independent prognostic value of the seven m6A-related lncRNAs prognostic signature

**Fig. 5:**
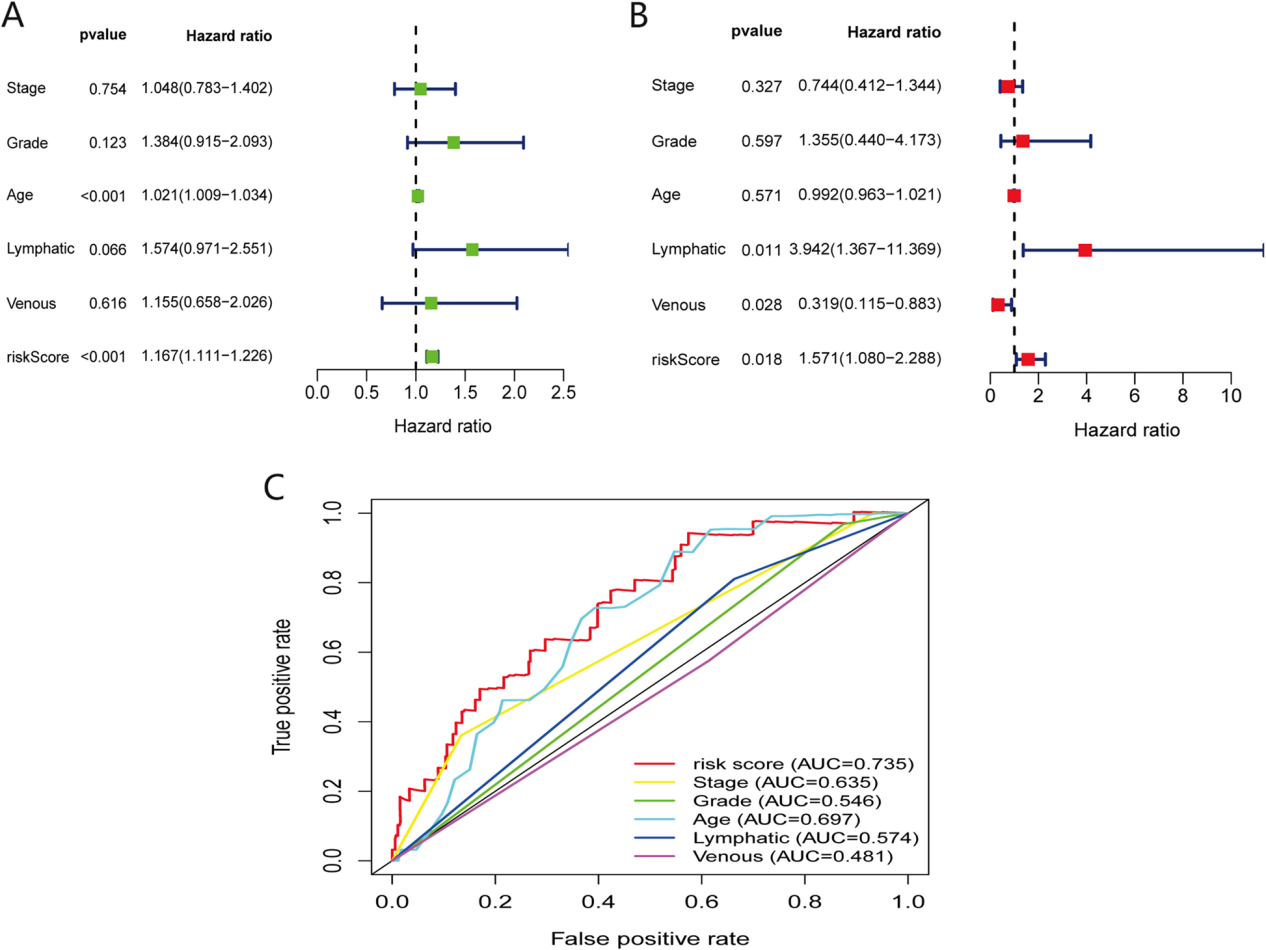
Univariate and multivariate Cox regression analyses based on the risk score and other clinical features in TCGA-OV dataset. **A** Univariate Cox regression analyses. **B** Multivariate Cox regression analyses. **C** ROC curve analysis of the risk score and other clinical features

Univariate and multivariate Cox regression analyses were performed in the TCGA-OV dataset to determine whether riskscore was an independent prognostic factor for OC patients. Univariate Cox regression analyses indicated that the riskscore was significant associated with the prognosis of OC patients (HR = 1.167, 95%CI = 1.111 ~ 1.226, *P* < 0. 001, Fig. 5A). Multivariate Cox regression analyses revealed that riskscore remains a significant prognostic factor after controlling for other confounders (HR = 1.571, 95%CI = 1.080 ~ 2.288, *P* = 0. 018, Fig. 5B), demonstrating that riskscore could be used as an independent prognostic factor of OC patients. The ROC curve showed that the prognostic ability of the riskscore was higher than the other clinical factors (Fig. 5C).

### Construction of the nomogram model based on the seven m6A-related lncRNAs prognostic signature

**Fig. 6:**
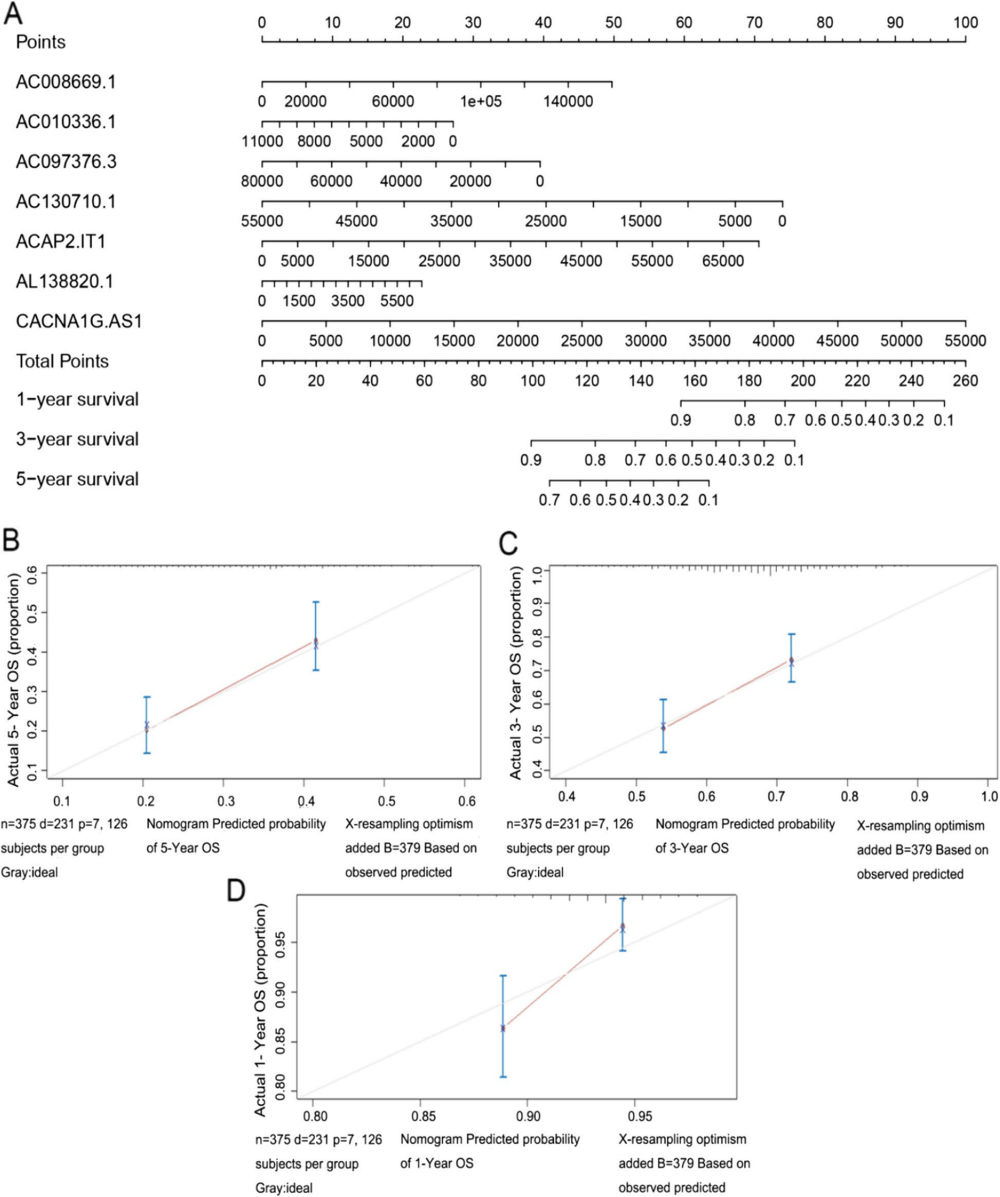
Construction of the nomogram model. **A** A nomogram for predicting the 1-, 3-, 5-year overall survival rates of OC patients. **B** The calibration curve at 1-year. **C** The calibration curve at 3-year. **D** The calibration curve at 5-year

A nomogram model was conducted based on the expression level of m6A-related lncRNAs prognostic signature to predict the survival rates of OC patients at 1, 3, 5 years (Fig. 6A). The calibration curve at 1, 3, 5 years revealed that the actual and predicted survival rates was highly consistent, suggesting that the predictive performance of the nomogram model was power ((Fig. 6B, C and D).

### Construction of the ceRNA network related to the seven m6A-related lncRNAs prognostic signature

**Fig. 7:**
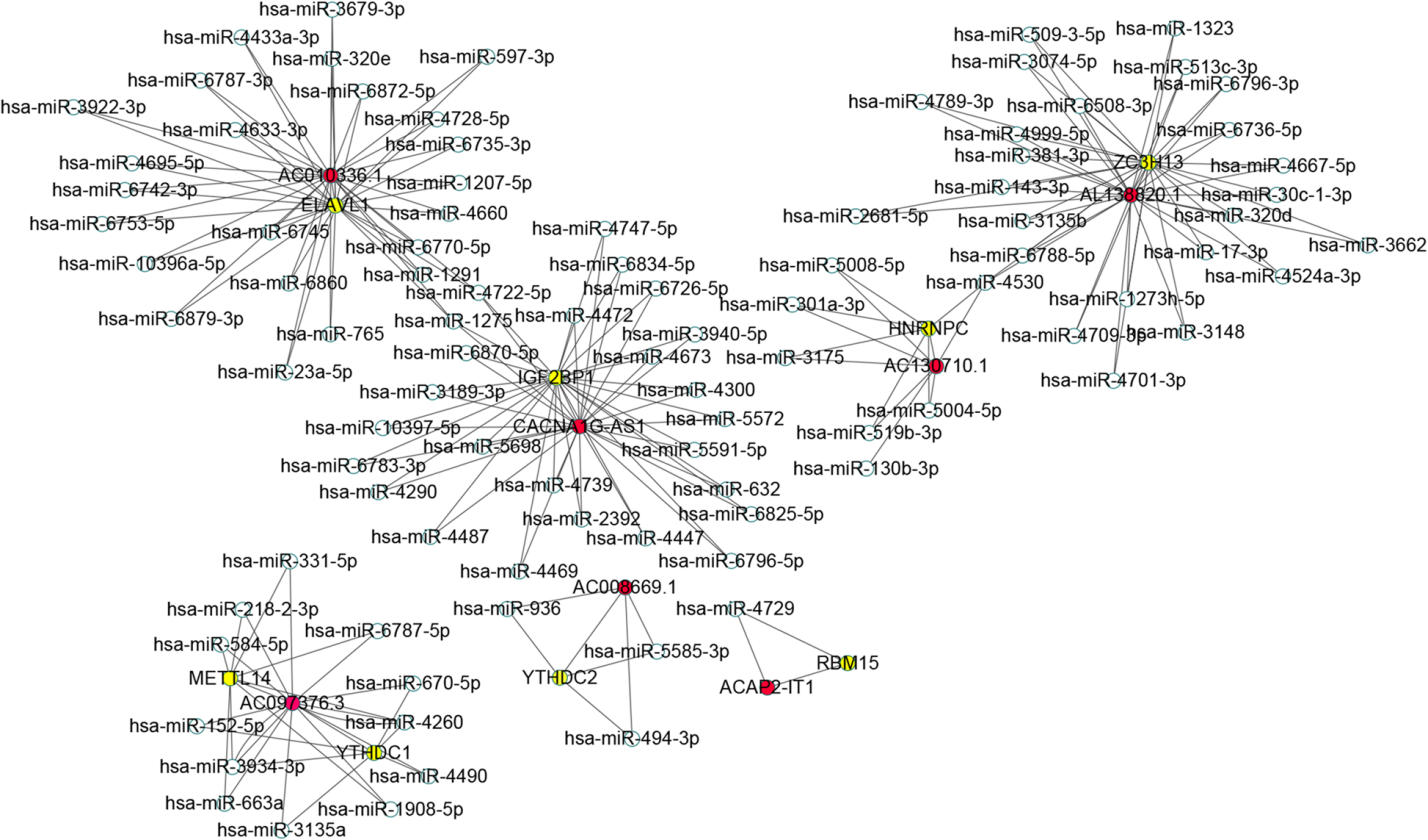
Construction of the ceRNA network associated with the the seven m6A-related lncRNAs prognostic signature. Red represents the seven m6A-related lncRNAs prognostic signature, white represents miRNA, yellow represents m6A regulators

A ceRNA network was constructed based on the seven m6A-related lncRNAs prognostic signature and the corresponding eighteen m6A regulators (Table 4). We obtained the microRNA (miRNA) interacted with the seven m6A-related lncRNAs from miRDB online website. Then, the miRNA interacted with the eighteen m6A regulators was obtained from miRWalk online website. After intersecting the predicted miRNAs, a ceRNA network including seven m6A-related lncRNAs, eighteen m6A regulators and two hundred miRNAs was obtained and visualized by cytoscope software (Fig. 7).

**Table 4:** Seven m6A-related lncRNAs prognostic signature and the corresponding eighteen m6A regulators

GENE1	GENE2	P	R
AC130710.1	HNRNPC	1.59E-21	0.462904
AC097376.3	YTHDC1	3.58E-21	0.459273
AL138820.1	ZC3H13	8.69E-20	0.444521
CACNA1G-AS1	IGF2BP1	9.04E-20	0.444332
AC010336.1	ELAVL1	1.25E-19	0.44279
AC097376.3	METTL14	4.99E-17	0.412807
AC008669.1	YTHDC2	1.11E-16	0.408546
ACAP2-IT1	RBM15	1.31E-16	0.407682

## Discussion

TCGA-OV dataset including 379 OC patients was used as training dataset to identify the prognostic significance of m6A-related lncRNAs in OC. 29 m6A-related lncRNAs with prognostic value were selected and seven of them were used to conduct a m6A-related lncRNAs prognostic signature for predicting the prognosis of OC patients. The patients in TCGA-OV dataset were divided into high- and low- risk groups based on the median riskscore, and the patients in high- risk group have poor outcome. Multivariate Cox regression analyses revealed that the riskscore was an independent prognostic factor for OC patients. We then conducted a nomogram based on the expression level of the seven m6A-related lncRNAs prognostic signature to predict the survival rate of the OC patients. Finally, a ceRNA network including seven m6A-related lncRNAs, eighteen m6A regulators and two hundred miRNAs was acquired to investigate the potential mechanisms of the m6A-related lncRNAs involed in OC. Reviewing previous studies, we found an article consistent with the purpose of our analysis [17]. Jianfeng Zheng etc. randomly divided the TCGA-OV dataset into training or validation dataset at a ratio of 3:7. In our study, TCGA-OV dataset was used as the training dataset, while GSE9891 GSE26193 dataset as well as 60 clinical specimens were used as the validation dataset. Jianfeng Zheng etc. selected 129 m6A-related lncRNAs based on the screening criteria *p* < 0.01 and |R|> 0.4, while our study aquired 275 m6A-related lncRNAs according to the screening criteria *p* < 0.001 and |R|> 0.4. Jianfeng Zheng etc. conduct a four m6A-related lncRNAs prognostic signature (AC010894.3, ACAP2-IT1, CACNA1G-AS1 and UBA6-AS1) for predicting the prognosis of OC patients based on the training dataset, while we built a m6A-related lncRNAs prognostic signature containg seven lncRNAs. ACAP2-IT1 and CACNA1G-AS1 was included in the m6A-related lncRNAs prognostic signature of two analyses. Jianfeng Zheng etc. found that the prognostic signature was confirmed to show completely opposite prognostic value in training dataset and validation dataset, while our prognostic signature was successfully confirmed in the validation dataset. Jianfeng Zheng etc. Superior to our analysis is that they further analyzed the differences of immune cell infiltration and chemotherapy drugs between high-and low- risk groups as well as the effect of CACNA1G-AS1 on ovarian cancer cell proliferation. The reason for some differences in the results between the two studies maybe that the sample size of the training dataset and screening criteria for m6A-related lncRNAs were different.

Numerous studies have shown that m6A regulators might play an important role in the malignant progression of cancers. Positively controlled by METTL3, LINC00958 promotes the tumorigenesis for breast cancer by regulating the miR-378a-3p/YY1 axis [18]. Jie Shen et al. reported that YTHDF2 promotes the proliferation of endometrioid endometrial carcinoma by increasing the degradation of lncRNA FENDRR [19]. Xiangrui Meng et al. have shown that m6A mediated overexpression of LINC00857 promotes the progression and tumorigenesis of pancreatic cancer through the regulation of miR—150–5 p/E2F3 axis [13]. LncRNA MALAT1 acts a oncogenic role in thyroid cancer by regulating the miR-204/IGF2BP2/m6A-MYC axis [20]. We can see that m6A regulators and lncRNAs can promote the progression of cancer through interaction from the above literature review. However, the potential mechanisms of m6A regulators involved in the lncRNAs-dependent OC pathogenesis remains unclear. In our study, we explored the potential interaction between the m6A regulators and lncRNAs through the construction of the ceRNA network, but more experimental studies are still needed to verify our conjecture in the future.

A seven m6A-related lncRNAs prognostic signature (AC008669.1, AC010336.1, AC097376.3, AC130710.1, ACAP2-IT1, AL138820.1 and CACNA1G-AS1) was conducted for predicting the prognosis of OC. Among them, AC008669.1, ACAP2-IT1, AL138820.1 and CACNA1G-AS1 was associated with poor prognosis of OC, while AC010336.1, AC097376.3 and AC130710.1 was protective factor for the prognosis of OC. CaCNA1G-AS1 has been reported to be associated with the malignant progression of liver cancer, rectal cancer, and non-small cell lung cancer [21–23]. In addition, other six m6A-related lncRNAs have not been reported yet. This demonstrates the novelty of our study and encourages us to continue to validate our findings in vitro and in vivo.

This study had some limitations. Firstly, we could not determine whether the seven selected m6A-related lncRNAs prognostic markers would be suitable for measuring in blood samples due to the sample tissues came from decidual tissue rather than blood. Secondly, ceRNA network of our research are only predictions and need to be verified by basic science and clinical studies. Finally, the number of OC samples in TCGA and GEO databased were limited, we need verify our research results using more other datasets.

## Conclusions

In conclusion, our research identified a seven m6A-related lncRNAs prognostic signature as a independent prognostic factors to predict the prognosis of OC. The seven m6A-related lncRNAs prognostic signature might act as prognostic markers and new therapeutic targets of OC.

## Supplementary Information

**Additional file 1.**

**Additional file 2.**

## Data Availability

The datasets generated and/or analysed during the current study are available in the TCGA (https://portal.gdc.cancer.gov/) and GEO (https://www.ncbi.nlm.nih.gov/) repository.

## Abbreviations

lncRNAs: Long non-coding RNAs
OC: Ovarian cancer
TCGA: The Cancer Genome Atlas
GEO: Gene Expression Omnibus
LASSO: Least absolute shrinkage and selection operator
EMT: Epithelial-to-mesenchymal transition
cAMP: Cyclic adenosine monophosphate
K-M: Kaplan–Meier
ROC: Receiver-operating characteristics
AUC: Area under the curve
