# Supplementary material for: Identification and validation of a seven m6A-related lncRNAs signature predicting prognosis of ovarian cancer
Source: BMC Cancer. 2022 Jun 8;22:633. doi: 10.1186/s12885-022-09591-4 (PMC9178823; doi:10.1186/s12885-022-09591-4)
Supplement: Supplementary file 1 — Additional file 1. [file 12885_2022_9591_MOESM1_ESM.docx]

Supplementary Table 1: Clinical information of the 60 OC samples.

| Characteristics | n |
| --- | --- |
| Age(year) |  |
| ≤55 | 28 |
| >55 | 32 |
| Lymph node metastasis |  |
| Positive | 34 |
| Negative | 26 |
| FIGO stage |  |
| I-II | 25 |
| III-IV | 35 |
| Tumer size（cm） |  |
| ≤10 | 36 |
| >10 | 24 |
| CA125 level（U/mL） |  |
| ≤300 | 37 |
| >300 | 23 |
| Survival state |  |
| Alive | 21 |
| Dead | 39 |
