# Supplementary material for: Identification and validation of a seven m6A-related lncRNAs signature predicting prognosis of ovarian cancer
Source: BMC Cancer. 2022 Jun 8;22:633. doi: 10.1186/s12885-022-09591-4 (PMC9178823; doi:10.1186/s12885-022-09591-4)
Supplement: Supplementary file 2 — Additional file 2. [file 12885_2022_9591_MOESM2_ESM.docx]

**Supplementary Table 2:** The sequences of primers used for RT-qPCR**.**

| Gene | Sequences of the primers |
| --- | --- |
| AC130710.1 | F:5'TGGAGGAAGGAACAGGCAGAGAG3'  R:5’ GAGGCACCACTTCAATAGGTCACTG3’ |
| AL138820.1 | F:5' AAAGATGCCCAAGGACCAGATGATG 3’  R:5’ TCTGAGATTGCTTGCCTGCTGATG 3’ |
| CACNA1G-AS1 | F:5' CAATGGGACGACAGTGGCAAGAG 3’  R:5’ CCCTTCACACGGTCACACATAGC 3’ |
| AC010336.1 | F:5’ TGCTTTAGACGGCTTTCGCTGAC 3’  R:5' TCTACACGCTCTACTGGCTCTTCC 3’ |
| AC097376.3 | F:5’ GAAATGGGCCTGGCTGTCGTATC 3’  R:5' ACTACTCCGCTCCAAGGTGACTC 3’ |
| AC008669.1 | F:5'AGGAAGGGAGGTTGCTGGTAGTTAG3'  R:5’ ACATAAGGAAGTGTGTGTCGAAGGG3’ |
| ACAP2-IT1 | F:5'CATGGCAGCATATTCTGGGTTGTTG3'  R:5’ AGGAAGTTGCCACCTTCACATACAC3’ |
| GAPDH | F:5’ CAAGGCTGTGGGCAAGGTCATC 3’  R:5’ GTGTCGCTGTTGAAGTCAGAGGAG 3’ |
